# Functional Effects of Dietary Grape By-Products on Rabbit Health, Performance, and Meat Quality

**DOI:** 10.3390/ani16040676

**Published:** 2026-02-21

**Authors:** Emmanuel O. Oladejo, Olivier Munezero, Nathaniel F. Ogunkunle, Barbara Still, Adam Handy, Yinka O. Adeyemo, Mark W. Murphey

**Affiliations:** 1 School of Agricultural Sciences and Forestry, Louisiana Tech University, Ruston, LA 71272, USA; 2 Department of Animal Science, University of Nebraska-Lincoln, Lincoln, NE 68583, USA; 3 Department of Food and Animal Sciences, Alabama A&M University, Normal, AL 35762, USA; 4 Department of Animal Nutrition, Federal University of Agriculture, Abeokuta 110111, Ogun State, Nigeria

**Keywords:** rabbit nutrition, grape by-products, functional feed, health, performance, meat quality

## Abstract

The increasing global demand for animal-source protein underscores the importance of sustainable livestock systems, including commercial rabbit production. Rabbits are valued for their short production cycle, high prolificacy, efficient feed utilization, and favorable meat quality. In parallel, agro-industrial by-products are being explored as functional feed ingredients to improve efficiency and reduce environmental impact. Grape by-products (GBPs), including pomace, seeds, stalks, and derived extracts, are rich in polyphenols, dietary fiber, and lipid bioactives with potential physiological benefits. This scoping review synthesizes current evidence regarding the effects of GBPs on rabbit health, growth performance, nutrient digestibility, reproductive function, and meat quality. Available studies indicate that moderate dietary inclusion can enhance antioxidant status, support immune function, modulate gut fermentation, and improve meat oxidative stability without consistently compromising growth performance when appropriately formulated. Benefits appear particularly evident under environmental stress conditions. However, outcomes vary according to inclusion level, processing method, and physiological stage, and excessive inclusion may impair nutrient utilization due to fiber and tannin content. Overall, this synthesis identifies functional mechanisms, practical feeding considerations, and key knowledge gaps to inform evidence-based application of GBPs in sustainable rabbit nutrition.

## Simple Summary

Rabbit production is valued for efficiency and high-quality meat but faces challenges related to health, feed utilization, and meat stability. Meanwhile, the wine industry generates substantial grape by-products (GBPs), including pomace, seeds, skins, and stalks, which are rich in bioactive compounds yet underutilized. This review synthesizes current evidence on the functional roles of GBPs in rabbit nutrition beyond nutrient supply. It evaluates effects on growth performance, blood health and immune status, growth performance, reproduction, and meat quality. Moderate inclusion levels generally enhance antioxidant capacity, support physiological resilience (especially under heat stress), and improve meat oxidative stability without consistently impairing growth or digestibility. However, responses depend on by-product type, processing method, inclusion level, and production stage. Constraints include high fiber, tannins, compositional variability, and safety considerations. This review identifies practical inclusion ranges, mechanistic insights, and research gaps to support sustainable integration of GBPs into rabbit feeding systems.

## 1. Introduction

Rabbits (*Oryctolagus cuniculus*) are commonly classified as monogastric herbivores with a well-developed cecum adapted for extensive microbial fermentation, a feature that distinguishes them from other monogastric species [1]. A defining characteristic of rabbit digestion is cecotrophy, which enables the complete utilization of nutrients derived from cecal fermentation [2,3]. This unique digestive adaptation enhances nutrient efficiency and contributes to the suitability of rabbits as meat-producing animals. Rabbits are known for short production cycles, high prolificacy, efficient feed utilization, and desirable meat quality characterized by low fat content, high protein levels, and a high proportion of unsaturated fatty acids [4]. Globally, rabbit production is most prominent in Asia, accounting for approximately 71% of total output, followed by Europe and Africa [5]. As global demand for animal-source protein continues to increase, rabbit farming represents a potentially sustainable option, particularly in developing and underdeveloped regions, due to rabbits’ adaptability to local feed resources and relatively modest production requirements [6].

In parallel, the winery industry, largely centered on grape processing, generates substantial quantities of by-products with potential applications in livestock feeding, although this potential remains underutilized. Despite this opportunity, grape by-products (GBPs) are occasionally incorporated into commercial rabbit diets primarily as fibrous feed ingredients when they are locally available, competitively priced, and meet safety standards such as mycotoxin limits. However, limited adoption persists due to variability in nutrient composition, seasonal availability, drying and storage costs, and regulatory uncertainty in some regions. Grape by-products, particularly grape pomace composed of seeds, skins, and stalks, have been shown to possess both nutritional and functional properties relevant to animal production [7,8,9], yet their application in livestock systems remains relatively underexplored. GBPs are rich in bioactive compounds associated with immunomodulatory [10], antioxidative [11], anti-inflammatory [12], and anti-helminthic [13,14]. These functional properties may help mitigate some of the adverse effects of antinutritional factors by supporting antioxidant defenses, gut integrity, enzymatic activity, systemic health, and overall animal performance. In addition to their bioactive components, GBPs retain appreciable amounts of nutrients, including fiber (17.3–88.7%), carbohydrates (12.2–40.5%), proteins and amino acids (3.6–14.2%), lipids (1.1–13.9%), and minerals (1.7–9.1%) [7,15,16] underscoring their potential value as alternative feed resources in animal nutrition.

Against this background, the present scoping review aims to synthesize existing evidence on the dietary incorporation of GBPs, including grape pomace, grape seeds (-meal, -oil, and -extracts), grape skins, and grape stalks, in rabbit production systems. Specifically, this review maps the current body of knowledge, identifies research gaps, and discusses potential applications, as well as intrinsic and extrinsic limitations associated with GBP and their bioactive compounds’ nutritional and functional value, respectively. Practical considerations and strategies for integrating GBPs into the diets of rabbits and other monogastric animals are also addressed. Through this synthesis, the review seeks to clarify the physiological effects of GBP supplementation on rabbit health, growth performance, productivity, and meat quality, thereby providing a structured foundation for future research and application.

## 2. Review Methodology

A comprehensive literature search was conducted using Google Scholar, PubMed, Scopus, and Web of Science. For studies directly related to rabbit nutrition and production, the search was restricted to articles published between 2008 and 2025 to capture both foundational and recent evidence, given the comparatively smaller body of published research for rabbits relative to other monogastric species. Search terms were applied in various combinations and included: “grape by-products,” “grape pomace,” “grape skin,” “grape seeds,” “grape stalks,” “grape by-products bioactive compounds,” “polyphenols,” “lipid bioactives,” “dietary fiber,” “rabbit,” “growth performance,” “blood and immune status,” “gut or intestinal health,” “reproductive performance,” and “meat quality.” Reference lists of relevant articles were also screened to identify additional studies of relevance with no specific period.

Data from included peer-reviewed original research and review papers were charted qualitatively and organized thematically according to major outcome domains, including systemic and intestinal health, growth performance, nutrient digestibility, reproductive performance, and meat quality. Despite fundamental physiological differences between rabbits and other monogastric species such as chickens and pigs, insights from these models remain valuable for identifying conserved biological mechanisms underlying digestive function. Rather than quantifying effect sizes, this scoping review synthesized findings descriptively to map the current evidence landscape. Knowledge gaps were identified based on limited data availability, inconsistent findings, insufficient exploration of underlying physiological mechanisms, and the absence of standardized processing methods or dosing strategies for grape by-products in rabbit diets. These criteria guided the narrative synthesis and thematic organization of the reviewed literature.

## 3. Some Valuable Nutraceuticals in Grape By-Products

### 3.1. Polyphenols

Polyphenols are plant-derived compounds broadly classified into flavonoids and non-flavonoids, with the latter group encompassing phenolic acids, stilbenes, tannins, and lignans [17]. The polyphenolic profile of grape by-products varies considerably depending on grape variety, plant fraction, cultivation conditions, and technological processing methods. Polyphenols are distributed across grape seeds, skins, and stalks [18], with total extractable phenolic content in grape pomace estimated to be approximately 60–70% in seeds, 30–35% in skins, 5–8% in stalks, and less than 10% in pulp [19,20].

A growing body of research has demonstrated that grape-derived polyphenols confer multiple health-related benefits in animals and may influence meat quality through their antioxidative [21,22], anti-inflammatory [22,23], antimicrobial [24], and antiproliferative [25] properties, positioning them as promising nutraceuticals or functional feed additives. However, not all polyphenols exert exclusively beneficial effects. Tannins, a subclass of polyphenols commonly regarded as antinutritional factors, represent a major constraint to the inclusion of grape by-products in monogastric diets [26], thereby necessitating careful consideration of inclusion levels and processing strategies.

For polyphenols derived from grape by-products to elicit positive physiological effects, they must be bioavailable within the biological system. Evidence from recent studies on grape skin polyphenol bioavailability indicates that absorption varies widely among individual compounds. In one report, only one of nine anthocyanins evaluated—malvidin-3-O-glucoside—was detectable in circulation, and at a low bioavailability of approximately 1% [27]. In contrast, simpler polyphenolic compounds, including gallic acid (a phenolic acid) and kaempferol-3-glucoside (a flavonol), were absorbed at substantially higher rates. These findings collectively suggest that: (1) grape skin polyphenols differ markedly in their bioavailability; (2) some polyphenols are absorbed systemically and may contribute to host-wide health effects, whereas others remain within the gastrointestinal tract, where they may exert localized actions related to gut health and microbial modulation; and (3) polyphenols from grape by-products may function as gut-active compounds capable of influencing inflammation and metabolic processes through microbiota–host interactions.

Supporting this concept, phenolic extracts derived from grape by-products have been shown to be partially bioavailable and associated with improvements in lipid metabolism in rodent models, an important indicator of cardiovascular health [28]. In the same study, the microbial-derived metabolite 3,4-hydroxyphenylacetic acid was detected in the plasma, liver, and urine of rats fed these extracts, further emphasizing the role of gut microbiota in transforming dietary phenolics into biologically active metabolites.

### 3.2. Lipid Bioactives

Grape seeds and grape pomace contain several major lipid constituents, including fatty acids, phytosterols, and tocols [29]. Among these, fatty acids (primarily linoleic acid, oleic acid, and palmitic acid) account for approximately 92–97% of the total fatty acid content. The fatty acid profile of grape pomace is characterized by a high proportion of polyunsaturated fatty acids, with linoleic acid (C18:2 n-6) identified as the predominant component. Across grape varieties, linoleic acid has been reported to constitute approximately 55–70% of total fatty acids [30,31], highlighting grape pomace as a rich source of essential n-6 fatty acids. Monounsaturated fatty acids are present at moderate levels, typically representing 16–17% of total fatty acids, with oleic acid (C18:1 n-9) as the dominant monounsaturated fatty acid [30,32,33]. In contrast, saturated fatty acids occur at lower concentrations, with palmitic acid (C16:0) being the most abundant saturated fatty acid [30]. Collectively, these profiles indicate that grape pomace is enriched with essential and bioactive unsaturated fatty acids, particularly linoleic and oleic acids, supporting its potential use as a functional feed resource. Nevertheless, the relatively high n-6/n-3 fatty acid ratio warrants careful consideration during diet formulation.

Despite the favorable lipid composition, the physiological activities of grape-derived lipid compounds, including antimicrobial, antioxidant, and anti-inflammatory effects, may be constrained in monogastric feeding systems due to the high lignified cell wall content and tannin levels present in grape by-products [34]. These structural and antinutritional factors can limit lipid release and utilization, thereby reducing bioavailability. Consequently, strategies such as enzymatic supplementation or pretreatment processes have been proposed to enhance the nutritional value and bioavailability of lipid components in grape by-products when incorporated into monogastric diets [34].

### 3.3. Dietary Fiber

It has been established that dietary fiber can provide a functional influence on gastrointestinal health and disease as well as eliminate mycotoxins from the body of monogastric species [35,36]. Fiber is classified into two major fractions: soluble fiber, including pectins, soluble hemicelluloses, and oligosaccharides, and insoluble fiber, comprising cellulose, hemicellulose, and lignin. In grape by-products, insoluble fiber represents the predominant fraction. Grape pomace typically contains approximately 50–60% total dietary fiber, largely derived from cellulose, hemicellulose, and lignin [37]. The fiber content of grape skins varies by grape variety, ranging from 51–56% by weight in red grapes to 17–28% in white grapes [38]. In addition, grapeseed oil retains non-digestible carbohydrates such as cellulose and pectin, while defatted grape seed residues have been reported to contain up to 74.3% total dietary fiber, including 67.1% insoluble fiber and 31.5% lignin [20,39]. Grape stalks, which constitute approximately 25% of grape pomace, are similarly rich in structural carbohydrates, containing 12–36% cellulose, 14–26% hemicellulose, and 23–34% lignin [40].

In rabbits, soluble fiber serves as an important substrate for hindgut microbial fermentation, thereby supporting gut function and microbial homeostasis [41]. Insoluble fiber, while less fermentable, contributes to intestinal motility and can help alleviate digestive disturbances; however, excessive inclusion may reduce dietary digestible energy and nutrient utilization [41,42]. Therefore, the dietary incorporation of grape by-products in rabbit nutrition requires careful consideration of both soluble and insoluble fiber fractions to achieve an optimal balance that supports digestive efficiency and overall systemic health.

## 4. Effects on Blood Health and Immune Status

Dietary bioactive components can alter hematological, biochemical, antioxidant, and immunological parameters [43,44], providing valuable insight into metabolic health, organ function and immune competence in animals [45]. In this context, polyphenol-rich diets have been shown to exert antioxidative, anti-inflammatory, and metabolic-modulating effects across various biological systems [46,47].

Several studies conducted under hot environmental conditions have demonstrated the therapeutic potential of grape by-products in alleviating heat stress in rabbits. For instance, Amer et al. [48] reported that dietary supplementation with 1.5% grape seed powder increased plasma total protein and globulin concentrations while reducing plasma total lipids and alanine aminotransferase (ALT) levels. Similarly, supplementation with 1 mL grape seed oil nano-emulsion per kilogram of diet resulted in reduced activities of ALT, aspartate aminotransferase (AST), lactate dehydrogenase (LDH), gamma-glutamyl transferase (GGT), as well as decreased uric acid, blood bilirubin, and lipid profile indices [49]. In addition, dietary inclusion of 200 or 300 mg grape seed extract (GSE)/kg diet elevated plasma total protein, albumin, and globulin concentrations, while lowering total cholesterol, triglycerides, and low-density lipoprotein levels in heat-stressed rabbits [50]. Taken together, these biochemical changes reflect improved physiological regulation, reduced metabolic stress, and enhanced liver function.

Beyond biochemical parameters, grape by-products have been shown to positively influence systemic antioxidant status. Supplementation with grape seed extract or grape seed powder increased both enzymatic and non-enzymatic antioxidant biomarkers in the blood of heat-stressed rabbits in a dose-dependent manner. Likewise, inclusion of 1 mL grape seed oil nano-emulsion/kg diet quadratically increased blood superoxide dismutase (SOD) and glutathione peroxidase (GPx) activities while decreasing malondialdehyde (MDA) and protein carbonyl concentrations. Notably, dietary supplementation with grape pomace powder also reduced blood MDA levels in rabbits fed a high-cholesterol diet [51]. These responses indicate the establishment of a robust redox defense system capable of neutralizing reactive oxygen species and protecting cellular components from oxidative damage.

Emerging evidence further suggests that grape by-products exert immunomodulatory and anti-inflammatory effects in rabbits. Recent studies have reported mitigation of heat stress through increased red blood cell counts, elevated immunoglobulin G and immunoglobulin M concentrations, and reduced inflammatory responses following dietary GBP supplementation [49,52]. These findings collectively signify enhanced systemic immune competence and resilience under stress conditions.

Overall, available evidence indicates that the dietary inclusion of grape by-products in rabbit nutrition supports improved physiological resilience, antioxidant capacity, and immune function. However, despite consistent reports of beneficial effects on blood biochemical, antioxidant, and immunological indices, there remains a paucity of information regarding optimal supplementation levels necessary to maximize benefits while avoiding potential toxicity or adverse impacts on systemic homeostasis. Table 1 summarizes current evidence on the effects of GBPs on blood health and immune status in rabbits. Future investigations should also explore the influence of grape by-products on blood cytokine signaling pathways and lysozyme activity, which serve as reliable indicators of systemic immune function and health status.

## 5. Effects on Growth Performance and Nutrient Digestibility

Growth performance is a primary indicator of animal health, productivity, and the economic viability of livestock production systems. Adequate dietary provision of nutrients supports metabolic processes that underpin growth efficiency and profitability. Historically, the use of sub-therapeutic in-feed antibiotics improved growth performance in rabbits by enhancing nutrient digestibility and feed efficiency; however, increasing concerns related to public health, food safety, and antimicrobial resistance have led to restrictions on their use [53,54]. Consequently, there is growing interest in identifying sustainable nutritional alternatives, including nutraceuticals derived from agro-industrial by-products, to support rabbit meat production [6].

Grape by-products have shown promise as functional feed ingredients for enhancing nutrient digestibility and intestinal health in rabbits [55,56]. Rabbits, as prolific hindgut fermenters, are particularly efficient at utilizing fibrous plant-based by-products. This advantage is attributable to their specialized digestive physiology, which enables effective fermentation of grape by-products when these materials are appropriately incorporated into the diet [6]. Cecal fermentation of grape by-products generates bioactive and highly bioavailable metabolites, including volatile fatty acids (VFAs), which contribute to improved intestinal function and overall animal performance [6].

Experimental evidence demonstrates that growth responses to grape by-product inclusion are dose dependent. Dietary inclusion of dehydrated grape pomace at 2% has been reported to improve feed intake, body weight gain, and fat digestibility in rabbits, while reducing crude protein digestibility without affecting nitrogen balance [56]. These findings suggest compensatory adaptations, whereby rabbits increased feed intake and enhanced fat utilization to maintain growth performance despite reduced protein digestibility. In contrast, Guemour et al. [57] observed that inclusion of grape pomace at 3% or 6% reduced feed intake, feed efficiency, and daily weight gain, with digestibility declining at the 6% inclusion level, while crude protein digestibility improved at 3%. Similar outcomes were reported at higher inclusion rates (20%), which reduced feed intake and feeding efficiency without altering final body weight gain [58,59]. Collectively, these findings indicate that antinutritional factors present in grape pomace may negatively influence digestibility and growth at higher inclusion levels, underscoring the need for targeted nutritional strategies to improve utilization.

Despite their relatively high lignin content, grape stalks have also been investigated as dietary components. Although generally considered nutritionally limiting [7,60], Costa-Silva et al. [7] reported that dietary inclusion of 5% or 10% fungus-treated grape stalks improved daily weight gain and feed efficiency in rabbits. These improvements occurred without significant changes in total apparent tract digestibility or intestinal morphology, suggesting that enhanced hindgut fermentation, rather than small intestinal digestibility, was responsible for the observed growth responses. The longer cecum and small intestine, along with increased total VFA concentrations, reflect greater fermentative capacity in rabbits, with VFAs serving as a key energy source that complements nutrients recovered through cecotrophy. Increased VFA production likely contributed additional metabolizable energy, thereby supporting growth.

Additional studies have reported improvements in rabbit growth performance following dietary supplementation with grape seed powder [48,61], grape seed extract [50], and grape seed oil [49], particularly under hot environmental conditions. These findings further highlight the potential of grape by-products to mitigate environmental or physiological stressors that negatively affect animal performance.

Overall, the utilization of grape by-products, including grape pomace, grape stalks, grape seed meal and extracts, and grape seed oil, in monogastric diets represents a promising avenue for functional feed innovation. Table 2 summarizes current evidence on the effects of GBPs on performance and nutrient digestibility in rabbits. While existing studies demonstrate beneficial effects on fat digestibility, hindgut fermentation, and growth performance, several knowledge gaps remain. Future research should focus on: (1) defining optimal inclusion levels of individual grape by-products or their combinations with other feed additives or supplements; (2) systematically evaluating how different valorization and processing methods influence digestibility and functional efficacy; and (3) elucidating the effects of polyphenol-rich, gut-active grape by-products on intestinal health and microbiota modulation. Addressing these gaps will be essential for the effective and safe integration of grape by-products into rabbit production systems.

## 6. Effects on Reproductive Health and Performance

**Figure 1:**
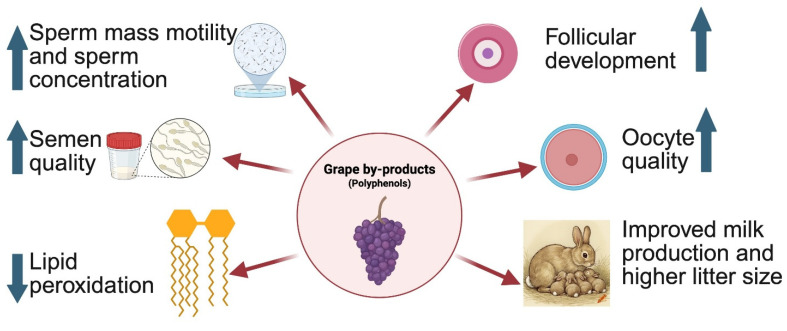
Influence of grape by-products on reproductive physiology and performance in rabbits. The drawing was created using Biorender.com. The pen in this figure indicates that information is not completely shown. The complete information is “ Improved milk production and higher litter size under stress”.

Nutrients play a crucial role in enhancing reproductive efficiency, as they modulate physiological changes and metabolic adjustments [62] and optimal outcomes depend on the provision of nutrients in appropriate proportions. With suitable processing and supplementation strategies, nutrients derived from plant-based materials, including crop residues and agro-industrial by-products, have demonstrated promising potential for improving reproductive performance in livestock systems [63,64,65]. Grape by-products are particularly rich in bioactive compounds capable of influencing key reproductive processes, such as sperm and semen physiology [66], follicular development, oocyte quality, endometrial function, and pregnancy outcomes [67] (Figure 1).

Emerging evidence suggests that dietary inclusion of grape by-products may enhance reproductive health and performance in rabbits. In bucks, dietary supplementation with 1% dehydrated grape pomace improved sperm mass motility and sperm concentration. Similarly, inclusion levels of 10% or 20% grape pomace in the diets of six-month-old bucks resulted in higher semen volume (up to 32% greater than unsupplemented controls) and improved sperm quality [68]. Supplementation with grape pomace was also associated with reduced lipid peroxidation and increased antioxidant activity in seminal plasma [68], thereby improving the protective and nutritive environment of spermatozoa [69]. Additional studies have shown that dietary supplementation with grape seed extract confers protective effects against reproductive toxicants. Hafsa et al. [70] reported that inclusion of 50 g/kg grape seed extract protected growing bucks from lindane-induced testicular structural and functional damage, while El-Ratel et al. [71] observed that administration of up to 125 mg/kg body weight of ethanolic grape seed extract improved testicular weight and semen quality in bucks, further supporting the reproductive benefits of grape-derived bioactives.

Although fewer studies have examined the effects of grape by-products on reproductive performance in does, available evidence indicates potential benefits. Dietary supplementation with a composite mixture of 0.5 g/kg grape seed and hydrolysable tannins improved antioxidant status during gestation, enhanced daily milk yield during lactation, and increased litter size and litter weight [72]. Despite these positive outcomes, the underlying mechanisms through which grape by-products influence reproductive function remain incompletely understood. Polyphenolic compounds present in grape by-products, including resveratrol and proanthocyanidins, are known to modulate reproductive physiology and pathology by interacting with multiple signaling pathways related to reproductive hormones, steroid receptors, and intracellular regulators of oxidative stress, cellular proliferation, and apoptosis [73]. Oxidative stress–induced inflammation has been implicated in disruptions of gametogenesis, sex hormone signaling, and the development of reproductive disorders [73,74,75]; however, bioactive phytonutrients with antioxidative and anti-inflammatory properties may help mitigate these adverse effects [76,77].

Beyond the limited number of studies conducted in rabbits, there is currently little information regarding the effects of dietary grape by-product inclusion on reproductive outcomes in other monogastric food animals, such as poultry and swine. Consequently, further research is warranted to elucidate the broader reproductive implications of this nutritional strategy. Future studies should investigate the effects of grape by-products on fertility and reproductive efficiency, including sexual behavior, testicular hemodynamics, epigenetic regulation, and gene expression associated with fertility, gametogenesis, and embryonic development, as well as the long-term health and performance of offspring.

## 7. Effects on Meat Quality

Meat quality is a critical determinant of market value, consumer acceptance, and profitability in animal production systems [1,78]. It is commonly evaluated using a combination of physical attributes, such as color, pH, and water-holding capacity [79,80]; chemical characteristics, including moisture content, protein concentration, and lipid composition [81]; sensory properties, such as flavor and juiciness [82], and microbiological or food safety indicators [83,84]. Accumulating evidence suggests that dietary inclusion of plant-derived by-products rich in nutrients and bioactive compounds can modulate these meat quality parameters in food-producing animals [85].

Grape bioactive compounds are partly bioavailable and can reach animal tissues, although their direct deposition in meat is often limited and variable. Clear effects have been observed in pigs and poultry with some studies reporting improved meat oxidative stability and fatty acid profiles rather than detectable phenolic accumulation [86,87,88,89]. High tannin content may restrict absorption, and in many cases, the antioxidant benefits appear to be indirect or mediated by metabolites rather than intact polyphenols [26]. Detection is challenging because tissue concentrations are typically very low and may fall below quantification limits, requiring highly sensitive analytical techniques such as HPLC, LC-MS/MS, or MSI [90,91]. Current research increasingly focuses on tracing polyphenol-derived metabolites which are more readily absorbed and more likely to reach muscle tissues than parent compounds.

Grape pomace contains high concentrations of PUFAs, comprising approximately 60–75% of the total fatty acid profile [92,93]. In addition, nearly 70% of grape polyphenols remain in the pomace following juice extraction during winemaking [93], underscoring their functional potential as a dietary supplement. In a recent study, supplementation of up to 10 g of grape pomace per rabbit per day did not alter the fatty acid profile of the *longissimus dorsi* muscle but significantly reduced thiobarbituric acid reactive substances by up to 60% [55]. This reduction indicates enhanced protection against lipid peroxidation, leading to improved oxidative stability and extended shelf life of rabbit meat.

Further evidence suggests that higher inclusion levels may also influence meat composition. Dietary inclusion of 20% grape pomace increased intramuscular fat content and modified the fatty acid profile of the longissimus dorsi muscle, particularly the polyunsaturated-to-saturated fatty acid ratio [59]. These changes highlight the potential of grape pomace to improve the physical and chemical attributes of rabbit meat, thereby supporting consumer acceptance. Notably, despite increased intramuscular fat, oxidative stability of minced hind leg meat stored for up to six days was not adversely affected. Concerns regarding elevated n-6/n-3 fatty acid ratios were also mitigated, as both atherogenic and thrombogenic indices were reduced. Consistent with these findings, Scerra et al. [94] reported that dietary supplementation with 10% grape seed effectively protected rabbit meat from lipid peroxidation during an eight-day storage period, further reinforcing the antioxidative role of grape-derived by-products in extending meat shelf life.

Despite these promising outcomes, the influence of grape pomace on rabbit meat quality remains incompletely characterized. Few studies have assessed consumer-oriented sensory attributes, such as taste, aroma, and tenderness, and most available trials are limited to the fattening phase. Consequently, supplementation effects of grape pomace throughout the production cycle on rabbit meat quality parameters remain unclear. Table 3 summarizes current evidence on the effects of GBPs on meat quality in rabbits. Further evidence from other animal products indicates that grape by-products and their extracts can enhance meat quality and safety when used as dietary supplements or marination agents, offering a biological framework for future investigations on rabbit meat. Future research could incorporate comprehensive meat quality evaluations that integrate physical, chemical, sensory, and microbiological analyses to provide a more holistic understanding of the effects of grape pomace on rabbit meat quality.

## 8. Limitations and Practical Strategies for Integrating GBPs into Monogastric Diets

### 8.1. Low Digestibility

Besides their nutritional value, GBPs contain substantial amounts of structural fiber and antinutritional compounds, particularly tannins, which can limit their inclusion in monogastric animal diets [7,34]. Excessive levels of these components have been associated with reduced feed intake [95], impaired nutrient digestibility and nutrient bioavailability consequent declines in production performance [7,96]. These constraints highlight the need for careful consideration of inclusion levels and processing methods when incorporating grape by-products into monogastric feeding systems.

Rabbits, however, possess a comparative advantage in utilizing fibrous feed ingredients due to their well-developed hindgut fermentative capacity and the practice of cecotrophy, which enhances nutrient recovery from plant-based materials [97]. Despite this physiological advantage, the dietary inclusion of grape by-products in rabbit nutrition must still be strategically managed to avoid adverse effects associated with excessive fiber or tannin intake. To address these limitations, several processing strategies have been explored. Enzymatic supplementation and fermentation have been shown to improve the digestibility and functional properties of grape by-products, thereby enhancing nutrient availability and the expression of bioactive effects [7,98].

### 8.2. Variability in Nutritional Profile

As is known, the nutritional and chemical composition of GBPs is influenced by multiple factors operating across the production chain, from grape cultivation to processing and storage. Grape maturity at harvest plays a critical role in determining the concentration of bioactive compounds, thereby shaping the overall chemical profile of the resulting by-products [99]. In addition, winemaking processes [100], grape type or varietal differences [38], specific valorization techniques applied during by-product processing [101,102], as well as post- processing storage conditions [103,104] influence the nutritional value of GPBs over time. Collectively, these factors represent key determinants of the compositional variability of GBPs and must be considered when evaluating their suitability and consistency as functional feed resources.

### 8.3. Mycotoxins

The presence of mycotoxins in feed resources can substantially limit their nutritional value and safety, particularly when grapes are exposed to mold contamination during cultivation, transportation, processing, and storage. Among these contaminants, Ochratoxin A (OTA), is the most frequently reported mycotoxin in grape pomace is the most widely reported mycotoxin in grape pomace [105]. OTA is primarily produced by *Aspergillus ochraceus*, *Aspergillus niger*, *Aspergillus carbonarius*, and *Penicillium verrucosum* [106] and has been associated with hepatotoxic, immunotoxic, nephrotoxic, and carcinogenic effects in animal studies [107,108]. These documented toxicological effects raise important safety concerns regarding the valorization and dietary use of grape pomace.

As comprehensively reviewed by Yu et al. [105], a range of mitigation strategies, including thermal pressure treatment, acidification, baking, and enzymatic interventions, have been evaluated for their capacity to reduce OTA concentrations in grape pomace. Together, these treatments were shown to enhance the safety of grape pomace relative to untreated material. Nevertheless, further investigation is required to determine whether combined or sequential application of these processing methods could achieve greater reductions in OTA levels.

In addition to OTA, other mycotoxins of concern have been detected in grapes and grape-derived products, including aflatoxins, fumonisin B2, and patulin [109]. These findings underscore the importance of rigorous quality control, appropriate processing, and continued research to ensure the safe incorporation of grape by-products into animal feeding systems.

### 8.4. Chemical Residues

Exposure to pesticide residues during grape cultivation, processing, and storage represents a potential source of contamination in grape by-products, with implications for both animal and human health [110,111]. Grapes are inherently susceptible to a range of pathogens and pests [112], which historically has led to the widespread use of chemical pesticides. However, advancements in modern grape production systems offer viable strategies to reduce the indiscriminate application of pesticides and limit the accumulation of residues in feed materials.

These mitigation approaches include the development and cultivation of pest-resistant grape varieties, increased reliance on bioinsecticides and biological control agents, and the adoption of advanced farm machinery and precision spraying technologies. Altogether, these practices aim to minimize pesticide inputs while maintaining crop protection, thereby reducing potential public health risks associated with pesticide residues in grape by-products [112,113].

## 9. Overall Conclusions

Current evidence indicates that GBPs, including grape pomace, seeds, extracts, oils and stalks, can provide functional benefits in rabbit production when included at appropriate levels. Moderate supplementation generally improves antioxidant defenses, supports immune competence, enhances stress resilience, and increases meat oxidative stability while maintaining acceptable growth performance and nutrient utilization. Reproductive benefits are promising but remain inconsistently documented. Observed effects are largely attributed to grape-derived polyphenols, dietary fiber fractions, lipid bioactives, and associated metabolites, which interact with gut microbiota and systemic redox pathways. Nevertheless, responses depend on by-product type, processing strategy, dosage, and animal physiological stage. Excessive inclusion or poorly characterized materials may reduce digestibility or introduce safety risks related to anti-nutritional factors or contaminants. When carefully formulated and quality-controlled, GBPs represent promising functional feed ingredients that contribute to improved rabbit health, product quality, and sustainable production systems.

## 10. Future Directions

Future research should prioritize standardized characterization of GBPs, including bioactive composition, fiber fractions, antinutritional factors, and potential contaminants. Rabbit-specific dose–response studies across production stages are required to define optimal inclusion ranges and evaluate long-term effects on health, performance, reproduction, and meat quality under physiologically challenging conditions. Mechanistic investigations linking polyphenol metabolism, microbiota modulation, immune signaling, and tissue-level responses are needed. Comprehensive safety assessments, including mycotoxin monitoring, alongside economic evaluations, should be incorporated. Expanded meat quality analyses covering oxidative stability, fatty acid balance, sensory attributes, and shelf life are also warranted. Addressing these areas will strengthen the scientific basis for integrating GBPs into sustainable rabbit feeding strategies.

## Figures and Tables

**Table 1:** Summary of current evidence on the effects of grape by-products on blood health and immune status in rabbits.

Age (Weeks)	Feeding Trial (Days)	Grape By-Products	Dosage	Physiological State	Indicators	Practical Implications	Reference
6	56	GSP	0.5–1.5%	Heat stressed	All doses showed ↑ plasma total protein & globulin, ↑ antioxidant enzyme activities, but highest with 1.5%. Also, ↓ plasma total lipids & ALT with 1.5%.	1.5% level appears more effective in improving hematological parameters & immune response.	[48]
6	56	GSO-NE	0.5–1.5 mL/kg diet	Heat stressed	All doses showed ↑ hematological values (RBC, Hb, Ht, platelets) but 1.5 mL/kg diet showed highest. 1&1.5 mL/kg diet showed ↑ antioxidant status, ↑ IgG & IgM, ↓ IL-6 & IFN-γ, and ↑ IL-10 & NO.	1.0 mL/kg optimally enhances hematological indices, humoral immunity, and antioxidant capacity while suppressing inflammation in heat-stressed rabbits, with minimal added benefit at 1.5 mL/kg.	[49]
6	56	GSE	100–300 mg/kg diet	Heat stressed	All doses showed ↓ plasma total lipid & MDA. 200 & 300 mg/kg diet ↑ plasma total protein &globulin but highest with 300 mg/kg diet. Antioxidant enzyme activities ↑ with 300 mg/kg diet.	300 mg/kg is the most effective dose for optimizing blood health and immune competence in stressed rabbits.	[50]
5	56	GSE	100–400 mg/kg diet	Heat stressed	All doses ↑ total serum protein, albumin & globulin, ↓ liver/kidney enzymes, ↑ IgM, IgG; ↑ antioxidant enzymes activities, and ↓ IL-4.	200 mg/kg diet shows optimal balance for blood health, antioxidant defense, and immune regulation, with limited added benefit beyond this level when cost efficiency is considered.	[52]

Note: ALT = Alanine aminotransferase; GSP = Grape seed powder; GSE = Grape seed extract; GSO-NE = Grape seed oil nano-emulsion; Hb = Hemoglobin; Ht = Hematocrit; Ig = Immunoglobulin; IFN = Interferon; IL = Interleukin; MDA = Malondialdehyde; NO = Nitric oxide; ↑ = Higher; ↓ = Lower.

**Table 2:** Summary of current evidence on the effects of grape by-products on growth performance and nutrient digestibility in rabbits.

**Age (Weeks)**	Feeding Trial (Days)	Grape By-Products	Dosage	Indicators	Practical Implications	References
12	140	Dehydrated GP	1–2%	2% inclusion ↑ ADFI, ADG, fat digestibility, ↓ OM and CP digestibility, with no effect on nitrogen balance.	1% has no effect on performance, while 2% can enhance performance but requires careful balancing of CP and energy digestibility due to fiber effects.	[56]
5	42	Sun-dried GP	3–6%	At 3% & 6%, growth rate ↓ but CP unaffected; OM digestibility ↓ at 3% & ↓ further at 6%.	Even low inclusion (3%) can impair growth and feed efficiency, indicating the need for careful dietary inclusion.	[57]
4	30	GP	100–300 g/kg diet	ADG is unchanged; G: F ↓ linearly with increasing GP, and apparent digestibility of CP & energy ↓.	GP can replace alfalfa hay, but high inclusion levels reduce nutrient utilization and feed efficiency.	[58]
5	30	GP	20%	G: F & carcass weight ↓; while ADG & live weight remain unaffected.	GP can be included at 20% without reducing live weight gain, but feed efficiency and carcass yield decline.	[59]
5	31	GS	5–10%	Untreated GS at both levels did not affect growth/feed efficiency, whereas fungal-treated GS (up to 10%) ↑ ADG, ↑ G: F & ↑ cecal fermentative activity.	Untreated grape stalks are safe but nutritionally limited, while fungi-treated grape stalks enhance growth performance and digestive efficiency.	[7]
6	56	GSP	0.5–1.5%	All doses ↑ growth but 1.5% showed highest feed efficiency despite ↓ ADFI.	1.5% GSP is best when feed efficiency is the priority.	[48]
9	28	GSP	50–150 mg/kg BW	50 & 150 mg/kg did not affect growth, whereas 100 mg/kg ↑ ADG and final BW.	100 mg/kg live BW effectively improves growth performance.	[61]
6	56	GSE	100– 300 mg/kg diet	300 mg/kg diet yielded highest ADG and feed efficiency.	300 mg/kg is the optimal level for improving growth performance and feed efficiency.	[50]
6	56	GSO-NE	0.5–1.5 mL/kg diet	1.0 and 1.5 mL/kg diet ↑ ADG and final BW.	1.0 and 1.5 mL/kg diet improve growth performance.	[49]

Note: ADFI = Average daily feed intake; ADG = Average daily gain; BW = Body weight; CP = Crude protein; G: F = Gain to feed ratio; GP = Grape pomace; GS = Grape stalk; GSP = Grape seed powder; GSE = Grape seed extract; GSO-NE = Grape seed oil nano-emulsion; OM = Organic matter; ↑ = Higher; ↓ = Lower.

**Table 3:** Summary of current evidence on the effects of grape by-products on meat quality in rabbits.

Age (Weeks)	Feeding Trial (Days)	Grape By-Products	Dosage	Indicators	Practical Implications	Reference
5	30	GP	20%	20% inclusion showed ↑ intramuscular %, ↑ PUFA/SFA ratio, reduced total VBN.	Meat is more palatable, nutritionally superior, and more stable during storage.	[59]
8	21	GP	5–10 g/day per head	GP ↓ lipid peroxidation (↓ TBARS) in a dose-dependent manner (10 g/day > 5 g/day), while fatty acid profile remained unchanged.	GP improves meat oxidative stability and shelf life without altering basic meat composition. 10 g/day provides stronger antioxidant protection than 5 g/day.	[55]
5	57	GS	10% diet	10% GS inclusion ↑ intramuscular fat %, ↑ MUFA (C18:1 cis-9), ↓ lipid peroxidation (↓ TBARS), ↓ n-3 PUFA, and ↓ n-6/n-3 ratio.	10% GS inclusion enhances juiciness and palatability and extends shelf life via better oxidative stability.	[94]

Note: GP = Grape pomace; GS = Grape seed; MUFA= Monounsaturated fatty acid; PUFA = Polyunsaturated fatty acid; SFA = Saturated fatty acid; TBARS = Thiobarbituric acid reactive substances; VBN = Volatile basic nitrogen; ↑ = Higher; ↓ = Lower.

## Data Availability

No new data were created or analyzed in this study. Data sharing is not applicable to this article.

## Abbreviations

The following abbreviations are used in this manuscript:

ALT	Alanine aminotransferase
AST	Aspartate aminotransferase
GGT	Gamma-glutamyl transferase
GPx	Glutathione peroxidase
HPLC	High-performance liquid chromatography
Ig	Immunoglobulin
LC-MS/MS	Liquid chromatography-mass spectrometry
LDH	Lactate dehydrogenase
MDA	Malondialedyde
MSI	Mass spectrometry imaging
PUFA	Polysaturated fatty acid
SOD	Superoxide dismutase
TBARS	Thiobarbituric acid reactive substances
